# How did we get here? A qualitative study of contributors to traumatic birth experiences in NICU parents

**DOI:** 10.1186/s40748-025-00236-5

**Published:** 2025-11-07

**Authors:** Roopa Gorur, Paris S. Ekeke

**Affiliations:** 1 University of Michigan School of Medicine, MI Ann Arbor, USA; 2 Divison of Neonatology, Department of Pediatrics, School of Medicine, University of Michigan, 1500 E Medical Center Drive, Ann Arbor, MI 48109 USA

**Keywords:** Birth trauma, NICU, Perinatal mental health

## Abstract

**Background:**

Birth trauma is a complex concept that encompasses experiences spanning the perinatal period, from prenatal care to delivery and postpartum care. Despite NICU parents being a high-risk population, there are limited studies examining interpersonal trauma in the NICU. Our study sought to explore the perspectives of NICU families on contributors to birth trauma and assess concordance with NICU staff knowledge on traumatic births.

**Methods:**

A multi-methods study was performed exploring the qualitative experience of postpartum parents with infants admitted to a level IV NICU. Each participant shared their prenatal, delivery, and postnatal experiences through a semi-structured, audio-recorded interview. Interviews were transcribed using HIPAA compliant software and verified by principal investigators for accuracy. Each principal investigator performed thematic analysis using the constant comparative method until saturation and consensus was reached.

Additionally, NICU Staff completed an anonymous survey soliciting baseline knowledge and attitudes regarding birth trauma. Perspectives from birthing parents and medical staff were compared.

**Results:**

Three themes contributing to birth trauma emerged among birthing parents (1) inadequate communication with the medical team (2) lack of support from trusted sources (3) fear of the unknown regarding their infants medical condition.

While 96% of medical staff acknowledged that implicit bias and interpersonal trauma contribute negatively to healthcare disparities, when probed about real life examples, 50% of staff were unsure if they had personally witnessed such events. Majority of staff believed “deviation from birth plans”, “prolonged hospitalization”, and “treatment decisions” would be the primary contributors to patients’ negative feelings about their birth. This was discordant with patient perspectives who frequently cited provider-patient interactions as the biggest contributor.

**Conclusions and relevancy:**

Interpersonal interactions play a huge role in patients’ perception of the birth experience. Despite this, good communication, access to support, and anticipatory guidance can be protective factors, but providers need more education to better understand birth trauma and how it can present. Trauma-informed care education among medical staff is needed to improve recognition of signs and symptoms of trauma responses and reduce re-traumatization of patients during the perinatal experience.

**Supplementary Information:**

The online version contains supplementary material available at 10.1186/s40748-025-00236-5.

## Background

Recent literature on disparities in perinatal outcomes suggests that the experience of the birthing parent is not homogenous [1]. Severe maternal morbidity and mortality remains high in the U.S. compared to other high-income countries, which includes psychiatric conditions such as birth trauma and post-traumatic stress disorder (PTSD) [2, 3]. Birth trauma is a complex concept that is difficult to define and encompasses a wide range of experiences [4, 5]. Traumatic birth experiences are described as overwhelming emotional distress directly related to interactions or events during childbirth [6, 7]. Between 28 and 40% of birthing parents around the world are reporting their birth experiences as traumatic and 1–6% go on to develop PTSD within the first year of giving birth [8, 9]. Racial and ethnic minority populations, parents with previous history of mental health disorders, and those who experience medical complications such as unexpected Cesarean section or postpartum hemorrhage are at increased risk of birth trauma [4, 10]. Causes of physical and emotional trauma are commonly referenced when describing birth trauma but interpersonal trauma is also a frequent contributor to traumatic birth experiences and remains poorly described [5, 6, 11, 12]. A study by Harris and Ayers also found that the strongest predictor of developing birth-related PTSD was interpersonal difficulties with care providers [13]. 

The concept of psychological trauma in the perinatal space often refers to perceived mistreatment of birthing parents and in addition to PTSD can also lead to anxiety, hypervigilance, and postpartum depression [14]. A systemic review by Bohren et al. described 7 different domains of medical maltreatment including: (1) verbal (2) physical or (3) sexual abuse (4) stigma and discrimination (5) failure to meet professional standards of care (6) poor rapport between women and providers and (7) health system conditions and constraints [15]. When asked about birth trauma, many patients described at least one experience that also fit into the maltreatment domains [16]. When clinicians were similarly asked about birth trauma, they often described maltreatment even when not specifically asked about this concept, supporting their interconnectivity [16]. 

Maltreatment and the associated trauma during pregnancy can have important implications for subsequent interactions with their medical team but also their infant’s medical team [17]. Multiple studies have shown that neonatal complications and NICU admission can be a large contributor to birth trauma, and the overall NICU environment can add additional stress [4, 15, 17]. However, there is sparse literature elucidating which elements of the NICU experience are highly associated with postpartum PTSD. Sharp et al. performed a mixed methods study which evaluated NICU-Specific Stress and its effect on the relationship between traumatic birth experience and post-traumatic stress symptoms. They found that 42% of birth parents mentioned distressing maternal emotional experiences (e.g., feeling guilty, sad, and anxious), 42% mentioned NICU characteristics (e.g. restricted access, frequent staff changes, noise and lights), and 33% mentioned dissatisfaction with their infant’s medical care all as contributors to birth trauma [15]. Despite NICU parents being a high-risk population, there are limited studies examining the dynamics of interpersonal trauma in the NICU. Many studies merely reference the stress of the medical complexity of NICU admission, but it is important to deepen our understanding in order to best support NICU parents [4, 17, 18]. Because of the deeply personal nature of the birth experience, it is imperative to center the voices and explore the lived experience of NICU birthing parents. The purpose of our study was to assess the major contributors of traumatic birth experiences in NICU birthing parents. Concurrently, we will examine baseline attitudes on birth trauma in NICU staff members to elucidate any discordance between families and staff perspectives as opportunities for future trauma-informed interventions.

## Methods

We conducted a multi-methods study of postpartum parents who had an infant currently admitted to our level IV NICU and staff in the NICU. This study was approved by our institutional IRB. Inclusion criteria for patient participation in the interviews included any birthing parent with an infant < 12 months old currently admitted in the level IV tertiary care neonatal intensive care unit (NICU). Inclusion in staff component of study requires staff member to be nurse, nurse practitioner, physician assistant or physician of our Level IV NICU. There were no exclusions. Participant recruitment flyers were placed around the unit and at each bedside with principal investigator contact information. If the birthing parent was present at the infant’s bedside, they were also approached. Once verbal interest was expressed, formal written informed consent was reviewed and signed by both the birthing parent and research team member conducting the interview. The interview was conducted anytime from 48 h to 12 months post-birth; scheduling was guided by participant preference. A single semi-structured interview was conducted with each participant which included asking questions about their prenatal, delivery, and postnatal experiences. A standard interview guide was co-created and generated by the University of Michigan Perinatal Psychology team.

Twenty-four interviews were conducted using an interview guide (see Appendix A) with open-ended questions and probes designed to assess the salient positive and negative elements of each period of their birth experience (prenatal, delivery, postpartum, and NICU). There was no set time limit on the interviews, and each interview was concluded when the participant answered all prompts and indicated that they have no additional comments. At the conclusion of the interview, a $15 gift card was distributed for their participation. Interviews were audio-recorded and transcribed using HIPAA compliant transcription software then manually verified by both principal investigators for accuracy. Transcripts were subsequently independently analyzed by each investigator for relevant themes using the constant comparative method and coded using NVivo software. Saturation of themes was reached by each investigator after analyzing all 24 interviews separately. After independent review, the investigators met to reconcile theme discrepancies and develop consensus on theme classification.

The second portion of the study involved soliciting baseline knowledge and attitudes on interpersonal bias from medical staff working in the neonatal intensive care unit (NICU) since it is a major contributor to traumatic birth experiences. An anonymous Qualtrics survey was sent via email to first line providers in the NICU, including registered nurses, neonatal nurse practitioners, neonatal physician assistants, neonatology fellows, and neonatology attendings. General questions about their beliefs about interpersonal trauma via unconscious bias then assessed their thoughts on major contributors of traumatic birth experiences from a patient perspective. Then staff responses were examined for concordance with actual participant responses.

## Results

Table 1 depicts demographic information of the 24 parent participants. Most participants underwent Cesarean sections in our institution (Inborn) with an average gestational age of 33.5 weeks. Average age of participant’s infant was 5.6 weeks with the youngest being 2 days old and oldest being 7 months old.

**Table 1:** Characteristics of participants’ birth experience

N = 24	N (%)
Race	
White	19 (79.2)
Black	2 (8.3)
Other	3 (12.5)
Mode of Delivery	
Vaginal	5 (20.8)
Cesarean section	19 (79.2)
Inborn	
Yes	19 (79.2)
No	5 (20.8)
Gestational age at birth	
< 28 weeks	2 (8.3)
28–33 weeks	11 (45.8)
34–36 weeks	6 (25)
>= 37 weeks	5 (20.8)

### Theme 1: communication with the medical team is inadequate and patient concerns are not being addressed

The most common contributors to a negative birth experience centered around issues with communication between the parents and the prenatal and delivery teams (OBGYN providers, L&D providers, and NICU providers respectively). Parents reported conversations with providers often “felt rushed”, were unbalanced with overwhelmingly negative information, and contained too much information given at once. Many of the parents cited getting conflicting information from different medical providers as a major source of stress and anxiety during their birth experience. With the complexity of obstetric care, clear messaging from their local obstetricians and the Maternal Fetal Medicine team and congruence of their messaging remains important to patients. Lack of clarity regarding their care and incongruence between medical teams bred mistrust between patients and providers. This mistrust is exemplified in the following quote taken from participant interviews.

*“And so I just wish that I would have been*,* like*,* handled better because at that point*,* like*,* I was really to a point where I was like*,* “hey*,* if you guys can’t figure out what’s going on with me*,* but I’m supposed to trust you guys to take care of my child who’s going to need services or surgeries*,* like*,* things*,* like*,* how is that going to work?”*

-Mother of an infant born with Gastroschisis at 36 weeks

Quote 2:

*And then when I did get…when they admitted me it was still kind of up in the air. Two of the doctors down here “Yea you’re ruptured” then it would be like no you’re not. Then it was you were. Then it wasn’t…so it was like which is it?*

*-Mother transferred to tertiary care hospital from local hospital due to concerns for complications of twin pregnancy with severe fetal growth restriction and PPROM of one fetus.*

Another salient feature parents recall that contributed to their negative birth experiences was the feeling that their concerns were not adequately addressed by the prenatal, birthing, and/or post-birth medical team (OBGYN providers, L&D providers, and NICU providers respectively). Birthing parents reported “not feeling heard” when it came to their physical and mental concerns. For example, they reported their pain not being adequately controlled during delivery was particularly traumatic. Parents highlighted dismissal of concerns from anesthesia and labor and delivery staff about appropriately placed epidurals and adequate pain control. In addition, birthing parents noted that providers were dismissive of their physical symptoms or concerns about their symptoms which made them feel alone and think that the medical team didn’t have their best interest at heart with reports of obstetric providers delaying treatment for headaches and elevated blood pressure readings as heralding signs of pre-eclampsia, preterm labor signs, bleeding concerns. Similarly, in the NICU, providers were noted at times to minimize parental concern about their infant’s vital sign instability leading to parents perceiving themselves as the sole advocate for their child’s well-being. These events gave the impression that their medical team was only interested in pushing their own agenda and not taking patients’ perspectives into consideration. The patient’s trust in their medical team became compromised and contributed to many patients feeling a loss of autonomy for themselves and their infant. Direct quotes from participants included below:

*Well, I got really upset, because, eventually, I felt different than I did the first time. I felt a pop in my back. I thought I was going to be paralyzed or something, ‘cause it is your back, and I started panicking. She’s like, “Oh, this is normal,” but it’s not for me.*

-Mother of 32-week preterm twins

Quote 2:

*Looking back*,* I really wish I would have insisted to do general anesthesia*,* ‘cause I’m still pretty traumatized by the whole experience and throwing up the whole time and sick.*

*-Mother describing her urgent Cesarean section experience and did not tolerate anesthesia well and had a panic attack*.

Quote 3:

*Like*,* where I was thinking about switching to a different hospital because I felt like I wasn’t being heard at that point and listened to.*

*-Mother describing multiple prenatal visits with complaints of pain and elevated blood pressures and kept getting sent home*.

Participants often didn’t feel validated by the medical team and noted there seemed to be a provider mistrust of patient experience. Whether it was a pain that felt different than normal or expected, a new symptom that patients felt were a part of bigger problem, or a provider ignoring a patient’s perception of medical therapies that have historically been successful or unsuccessful for them, such instances contributed to patients feeling dismissed and that their provider didn’t believe them about their own bodies.

*So they had me drive down here, and I was all prepared. Like, I got everything ready. Like, they said, you’re going to have your baby. Like, we get down here. And the one doctor I saw, um, told me I was lying. “That’s a really nice story you’re telling me but you’re not ruptured.*

- Mother of an infant born with Gastroschisis who ultimately went into preterm labor

### Theme 2: parents reporting a lack of support from familiar people triggered their anxiety

In the prenatal period, participants expressed worry about not having their support system involved in their pregnancy and delivery experience. Feeling alone when their spouse or family member could not be present led to immense pressure on the birthing parent that they were solely responsible for advocating for themselves and their infants.

There were also reports of anxiety if women did not have a previously established relationship with their care team. They noted having to make important medical decisions in collaboration with providers that they just met and had not seen in previous encounters felt anxiety-provoking. Parents also noted feeling unsettled if their care team expressed their care was too complex or made statements about how “they had never seen this before”. When thinking about the instances where their care had to be transferred from their local obstetrician to a quaternary center, participants expressed appreciation for the transparency about the need for higher level of care, but also noted feelings of abandonment in losing their “medical home” resulting in not knowing who to contact when even the most basic obstetric concerns arose.

*“Not through here*,* but once my regular OB knew that I was seeing the high-risk doctor here*,* they refused to see me. That was hard on me because then that meant all my care had to come from here and it’s an hour drive here and an hour drive back. That was a negative experience.”*

-Mother of 38-week infant admitted to the NICU

*So I just*,* I guess maybe I just misunderstood that the doctors I was seeing wouldn’t be the ones that delivered the baby. Got it. So it was a different*,* like*,* okay*,* I just met you and you’re going to cut me open*,* which is good. But I think that was a little different part of the delivery. It was*,* I barely*,* I didn’t know the doctor at all. I had just met him like five minutes before. Yeah.*

-Mother transferred to MFM service for delivery of her 27 week twins

Once in the NICU in the postpartum period, there was a lot of emphasis placed on their level of anxiety being tied to familiar staff taking care of their baby. Having a primary nurse for their baby was therapeutic for their hypervigilance and overall anxiety. Conversely, exposure to new medical staff that were unfamiliar with their infant often led to feelings that parents could not leave the bedside due to fear of missing a significant clinical change in their infant and the medical team not recognizing it. The weight of being the primary advocate for their infant felt overwhelming at times.

*I feel like now I have to be here and advocate and watch and every time I see [vital sign change] I have to like write it down so that way they know and it makes it uneasy going home.*

-Mother of 31-week infant admitted to the NICU

Quote 2:

*I get a little paranoid with that*,* or I may get a little bit an anxiety when I have to see a new face*,* a new nurse that doesn’t know my baby.*

- African American mother who has a 28 week infant in NICU

### Theme 3: fear of the unknown

Although parents acknowledge that pregnancy complications can be unexpected, there was often a feeling of being out of control when things came up that they felt unprepared for. Patients noted that there often was a feeling of being overwhelmed with new information.

*I was so surprised and startled by that information, I wasn’t even prepared to really talk about it in that session. That makes it tricky. Because here’s all the doctors, everybody paying 100% undivided attention on to you and your baby, and what questions can answer for you. Then sometimes, we do, we get startling information. I need time to process that.*

-Mother of a 33-week preterm infant

Some patients mentioned feeling like their medical team held back vital information about potential complications which led to resentment from losing the opportunity to mentally prepare themselves. The uncertainty of what would happen to them or their infants at times was described as “traumatizing” or “debilitating”. Participants often described their feeling of loss of control was often triggered well into the postpartum period and during their infant’s NICU stay.

*“It’s stressful like when you don’t know what it is…you don’t know had to read it. I’m not a nurse. I just see that it goes up and down*,* up and down. And when it doesn’t*,* I already know that that’s not good. It means that it’s either she’s not breathing or her heart’s not beating. And it*,* you know*,* sometimes there was like instances where they were like*,* oh*,* there’s another like beat in there. And I was like*,* oh*,* no. Is something wrong with her heart?*

-Mother of a 31-week preterm infant

Quote 2:

*Oh*,* I was scared. I was terrified because*,* one*,* this is my first pregnancy. Two*,* I don’t know what I’m doing. I’m doing it by myself. I did all this by myself. They’ve already poked*,* prodded at me*,* and everything else. I felt like garbage*,* and now I have to go get an epidural and get my baby cut outta me. It was scary*,* very scary.*

- Mother of 29 week preterm infant born via emergency Cesarean section

### Contributing factors to positive birth experiences

When considering the spectrum of themes, there were often counter positive contributors that were also identified. Generally, patients preferred communication that is bidirectional and allowed time for them to digest information and get follow up questions answered. Prenatal counseling and anticipatory guidance were preferred compared to rushed explanations when complications occurred. Patients often commented that it was difficult to come up with questions in the moment and appreciated it when the medical team came back in subsequent encounters to address additional concerns. Counseling that was well-received was thorough, anticipatory, and checked for patient understanding throughout the conversation.

*" I’ve set the tone now*,* like*,* so this is what we’re doing today*,* and what’s the big picture impact of that? I’ve started advocating for my understanding. That’s what I need in these conversations. That has helped a lot. The folks who have been with us now*,* that are often with us multiple days*,* they’re getting that that’s the case. Now they’re coming and prepared to talk about that and help to paint that picture better.”*

*- Mother of a 33-week preterm infant discussing how she navigates taking in information on NICU rounds*.

Families also leaned into the elements of emotional and mental support that were provided by the medical team and identified that as a salient contributor to how they viewed their birth experience. Validating their emotional stress, feeling heard (i.e. parents felt that their concerns were taken seriously by the infant’s care team), offering reassurance, or just being a familiar face and calm presence were cited as sources of decreasing anxiety. Parents often noted small gestures from the medical team such as holding their hand or walking with them often made them feel cared for as the birthing parent and gave them the feeling the team had their best interests at heart. Offering mental support for birthing parents and encouragement from the medical team for birthing parent to also attend to their own needs gave parents a sense that the team wanted the best for the maternal-infant dyad as a family unit and gave them a sense of comfort.

She was supposed to be the baby’s nurse, but she came and held my hand when I was getting my spinal and just talked me through it. And even though she had stuff that she was supposed to be doing, she just sat there to make sure I was okay before my mom came in and it just made a difference that she went that extra step like “hey calm down”, you know, “you gotta arch your back more or do this and you know just kept me calm. It was really just kind of nice.

- Mother of an infant born with Gastroschisis

When considering the care for their baby, parents often expressed a desire to be very involved in the hands-on care of their infant. From basic care needs such as feeding and changing their infant’s diapers to more complex needs such as placing nasogastric tubes and ostomy care, being an active member of their infant’s care team put parents at ease. Although this sentiment was noted frequently, there seemed to be a delicate balance between hands on care and hypervigilance. Many parents reflected on early feelings of not being able to leave the bedside due to discomfort with the care and need for advocacy for competent care. These anxious feelings seemed to reduce greatly with continuity of care among the team.

*The nurses that have chosen him for primary have been—it’s a relief when they’re here. They know him. I know that if I have to run out*,* if I have to do anything*,* I don’t even have to worry about it ’cause they know him so well.*

- Mother of a 31-week preterm infant

### Perspectives from staff

There were 50 total respondents, 74% of the respondents were bedside nurses while the remaining 26% were advanced practice practitioners and physicians. Of all respondents, 96% agreed that unconscious bias could negatively impact patient care and 92% endorsed awareness of research that inequitable health care delivery contributes to disparities in perinatal outcomes, but when asked if they had personally witnessed an instance of inequitable treatment, only 34% of respondents could recall one while 50% of respondents were unsure. Examples of patient maltreatment were elicited from staff and summarized in Table 2. When asked more generally their thoughts on which factors contributed most to patients’ perception of negative birth experiences, 78% of respondents believed details of the medical care, broken down as “deviation from birth plans”(34%), “prolonged hospitalization”(24%), and “treatment decisions”(20%) as the perceived primary contributors to patients’ feeling about their birth, while 20% of respondents believed provider-patient interactions would be the most common complaint from patients who reported a negative birth experience and 2% believed “lack of social support” would be a primary contributor.

**Table 2:** Staff perspectives qualitative experience with interpersonal trauma

“A woman came in with preterm labor with twins and the OR was complete chaos. No one knew the patient’s name or was talking to her. She was crying out in pain or perhaps fear. When I asked if she has anyone with her I was told I should ask triage because they also didn’t know that.” - NICU Physician	“I took care of a baby who was 27ish weeks with African American parents that were deemed “difficult.” The medical team kept rounding outside the room because they “didn’t want to deal with mom.” It was also difficult to get them to bedside to assess any concerns. The patient ultimately got NEC and passed away.” - NICU Registered Nurse
“I have witnessed many black mothers be talked about behind their door as less then due to assuming things about their situation and or that she can’t really be in that much pain. - NICU Physician	“Judgements are made based on ethnicity and socioeconomic factors and people assume the worst therefore are less engaged and champion these babies less” - NICU Registered Nurse
“A known heroin addict coming in with no prenatal care, unknown gestation. Her pain was uncontrolled and she was left to labor, alone, because there were other obstetrical emergencies happening at the same time” - Neonatal Nurse Practitioner	“I have felt in many instances that once a family is labeled as “difficult” for whatever reason, the team is less likely to respond as quickly when I call for a question or when parents want to have discussions.” - Neonatal Nurse Practitioner
“Meconium drug screens that are ordered based off of opinions and judgments of a person’s lifestyle vs actual facts or following policy.” - NICU Physician	“Provider asking a mom who her second caregiver was and assuming there wasn’t a dad involved. The baby was African American” - NICU Registered Nurse
“Many of our mother’s of color get deemed difficult. At times leading to less engagement with the family. It also seems like the lower SES patient population is very frequently here, potentially due to financial circumstances, but I have also had parents tell me they did not feel welcome and at time judged at bedside.” - Neonatal Nurse Practitioner	I’ve seen countless examples of our “difficult” families being dismissed or not taken seriously when it comes to concerns of their infants plan of care or NICU trajectory. - NICU Registered Nurse

## Discussion

Birth trauma is highly prevalent among parents in the neonatal intensive care unit. Often, providers considered medical complications in birthing parents or their infants to be the major contributors to having negative feelings about the birth experience. However, our study highlights the role of interpersonal trauma as a major cause of birthing parents’ negative experiences. At the time of interviews, their infant could be several weeks old but when asked broadly about their experience, they vividly recounted prenatal events from months prior as significant events in their journey. Although almost every participant (23/24), cited a provider-patient interaction as being impactful, very few attributed these interactions to unconscious bias. We theorize this is due to the racial composition of the cohort which was 80% White. We view this as a limitation in our ability to accurately capture the contribution of unconscious bias in interpersonal trauma. Despite this predominance, our cohort appeared to be representative of the patient population in our unit in terms of racial, gestational age, and inborn differentials. We did note a higher rate of Cesarean sections compared to our institutional data which may have skewed some participants overall perceptions of their perinatal experiences but very rarely did participants, in general, raise concern about their Cesarean section being unnecessary.

When reflecting on their experience, there appeared to be several reoccurring concepts that came across: disrespectful communication, feeling unsupported, and being uncomfortable with the uncertainty. With regards to communication, patients were looking for an honest assessment of the clinical situation and clinicians taking the time to clearly communicate their medical reasoning while still remaining responsive to patient concerns. Disrespectful communication could be overt, characterized by rude behavior, dismissive body language, or more insidious by disrespecting patient autonomy. Birthing parents often reported negative feelings if it appeared the medical team was holding back information for fear of upsetting them or not fully explaining the potential complications when medical decision making is required. This correlates with the feelings of loss of autonomy reported in previous studies [13, 16, 19]. When patients feel they are not given the opportunity to make a fully informed decision, they are robbed of the opportunity to make an autonomous decision free from clinician bias. Whether it is a decision about termination, resuscitation, delivery planning or vaccinations, it remains paramount that patients feel like they have a sense of control over their bodies and their infant’s bodies [16, 19]. The concept of feeling unsupported was also a recurring theme. Birthing parents often referenced acts of kindness that allowed them to feel that we are not only interested in taking care of their bodies but also their minds and emotions. No one should give birth alone and there were many instances reported where feeling alone was a major source of anxiety in this population. Small gestures such as holding a hand during a difficult time, positive affirmations, or offering external support services through social work and perinatal psychiatry made patients feel seen and heard were often reported and the most impactful. Lastly, being uncomfortable with uncertainty or the fear of the unknown was also frequently described. Whether NICU admission was expected or not, there were often descriptions of parents feeling debilitating anxiety about their infant’s diagnosis and the ultimate outcome of their birth course. Threats of maternal mortality or infant loss made a lasting impact on birthing parents and were noted to be motivating factors for hypervigilance in the NICU.

To understand how best to support our birthing parents, we must sincerely assess potential gaps and educate ourselves on the ways in which our words may traumatize our patients. The purpose of the staff surveys was to obtain a snapshot of the baseline knowledge of our staff in order to tailor future educational initiatives. When contrasting parent experience with staff perception, there was a clear discordance. Although most staff recognized unconscious bias as a source of differential care in theory, many were unsure of specific examples of how this may present in their clinical practice. From staff perspectives, many believed that deviation from the parent’s birth plan was the major contributor to birth trauma which was not supported by the patient responses which heavily focused on interpersonal interactions. This dichotomy of responses on the topic of traumatic birth experiences highlights a cognitive dissonance between patients and staff. It seems the medical community often conceptualizes birth trauma by events that happen to the birthing parent without sincere acknowledgement of how interpersonal trauma with medical personnel contributes to this perception [16]. This invalidation can have down-stream effects on the care of maternal-infant dyads. From a medical perspective, feeling dismissed by providers and experiencing medical trauma has the potential to impact how parents seek care in subsequent encounters [20]. Avoiding over-medicalization of subsequent birth experiences by opting out of prenatal care, the use of unlicensed midwives or providers, and engaging in home births are often attempts by patients to regain control of their birth experiences [21]. 

In addition to the previously described consequences on maternal mental and physical health, the effects of traumatic birth experiences often linger into their postpartum experience as well. Birthing parents often feel ill-equipped to process the negative feelings around their birth, and these feelings combined with the invalidation of their experiences from medical personnel leads to avoidant behaviors commonly seen in the NICU. To avoid dealing with their own emotional trauma, patients instead tend to become hypervigilant in their infant’s care. Although few parents in our study had insight into how their prenatal trauma reflected in their behaviors with their infant’s care team, many often reflected that being included in their infant’s care and being heard by their infant’s care team were anxiety-reducing behaviors. Dismissive communication and disrespect by the infant care team were noted to cause flashbacks of previous feelings of loss of control and not feeling heard which triggered parents’ “fight or flight” response and impacted their ability to calmly process their infant’s medical information. Labile, dysregulated behavior is often referred to in the literature as a “trauma response” and can present as extreme anxiety and hypervigilance often resulting in micromanaging of care, detachment from participating in infant care, and/or anger. This study had important implications for clinicians. First, the results of our study elucidate some salient features of the birth experience that be improved to minimize the risk of traumatization in an already vulnerable population. Efforts to incorporate warm handoffs when transferring care among obstetric providers, two-touch antenatal counseling model, and difficult conversation trainings are all worthwhile considerations to mitigate trauma and improve patient experience. Additionally, there is a benefit to staff experience as well. Secondary trauma from witnessing traumatic events and navigating challenging patient interactions contributes to burn out in perinatal providers with rates reported as high as 25–30% [22]. Dedicated efforts to integrate trauma-informed care practices into all labor and delivery and neonatal intensive care units would be impactful.

Overall, birth trauma remains underrecognized among medical professionals. Based on the findings from this study, many providers lacked awareness regarding the role interpersonal trauma plays in serious potential short- and long-term consequences on the birthing parents and their infants. Future education among medical staff should focus on the importance of interpersonal interactions and how to be transparent, empathetic, and express mutual respect when communicating with patients. It is imperative that we enhance education among medical staff to improve recognition of signs and symptoms of trauma responses and trauma-informed care to reduce re-traumatization of our patients throughout the entire perinatal experience.

## Supplementary Information

Supplementary Material 1.

## Data Availability

De-identified data available upon request.
