# Supplementary material for: How did we get here? A qualitative study of contributors to traumatic birth experiences in NICU parents
Source: Matern Health Neonatol Perinatol. 2025 Nov 7;11:38. doi: 10.1186/s40748-025-00236-5 (PMC12593826; doi:10.1186/s40748-025-00236-5)
Supplement: Supplementary file 1 — Supplementary Material 1. [file 40748_2025_236_MOESM1_ESM.docx]

Appendix A.

**Family Experience on Perinatal Care**

We are conducting a brief survey about your pregnancy and birth experience. We are interested in learning more about your delivery. Please answer the following questions to the best of your abilities. Your responses are completely anonymous and will not be communicated back to you or your baby’s doctors.

Self-identified race: _________________

What hospital was your baby born? _______________

What was the gestational age at birth (How many weeks were you?): __________

How was your baby born (Vaginal delivery or C-section)? _____________

***We want to know about your experiences with the health care you received during your pregnancy, at the time of your child’s birth and after you gave birth. We will go through each period, one at a time. I especially want to focus on your experiences with the health care professionals – this can include doctors, nurses, physician assistants, midwives, doulas, pregnancy educators, lactation consultants, and others with whom you interacted. Everyone’s experience is different, and our team hopes to use what we learn from all these interviews with women to give feedback and recommendations to health care providers that are truly based on what women need.***

***Tell us about your pregnancy:***

**1. Where did you receive your pregnancy care?** *(write name of hospital, practice, or provider; if more than one, write all) _______________________________*

a. Were you able to attend to all health appointments your health care providers recommended for you?

c. *(if the woman says no, say*☺ I get it. What were some of the things that got in the way of having some of your prenatal care appointments?

**2. Now, thinking about your providers more generally?**

*a.* Are there exchanges or instances that stand out in your mind as particularly helpful during your prenatal care? (*ask lots of details – who, when, what happened*)

b.Were there times when you felt your providers were not helpful, treated you poorly, or your needs were not addressed during your medical care? *If yes,* can you give me an example? (*ask lots of details – who, when, what happened*)

**5. What, if anything, about your prenatal care could have been better?** a. Is there anything else that you wish your prenatal care team would have done for you? And/or what would you wish for next time?

**II. BIRTH**

***Now we are going to switch gears a little bit and talk about the birth of your (son/daughter, name). I am interested in hearing about your experience when you arrived at the hospital and went into labor and how that all went.***

**1. Where did you deliver your baby (use baby’s name sometimes)?**

**2. Can you tell me about your delivery experience?**

**a.** *If delivered at hospital or clinic,* when were you admitted to the hospital or clinic? *If delivered at home –* when did labor start?

***b.*** Was your birth experience what you hoped/wished for? What could have been done differently?

**c.** Did you experience any complications during birth?

d**.** Were you expecting your baby to have to come to the NICU?

***3.* Was there a healthcare provider during your delivery experience that you felt especially comfortable with?** (*Include role of provider, if known) ______________________________________________*

**a.** In what way did this person make you feel supported during the birth and early postnatal care?

***4.* Now, thinking about your providers more generally?**

**a.** Did you feel supported by your health care team during the birth and early postnatal care?

**b.** *If yes,* what did they do to make you feel supported? *If not*, what could they have done to be more supportive?

**c.** Are there exchanges or instances that stand out in your mind as particularly helpful during your birth and early postnatal care? (*ask lots of details – who, when, what happened*)

**d.** Were there instances or situations when you felt your providers were not helpful, treated you poorly, or your needs were not addressed during your medical care? *If yes,* can you think of an example? (*ask lots of details – who, when, what happened*)

***5.* What, if anything, about your care around birth and delivery could have been better?**

***a.*** Is there anything else that you wish your birth health care team would have done for you?

**6. . Based on your most recent pregnancy experiences, what changes do you plan to make, if any, in subsequent pregnancies?**

After Birth

**Thinking about your providers more generally:**

a. Are there exchanges or instances that stand out in your mind as particularly helpful from the care you received after the birth of your baby? (*ask lots of details – who, when, what happened*)

b. Were there instances or situations when you felt your providers were not helpful, treated you poorly, or your needs were not addressed during your medical care? (*ask lots of details – who, when, what happened*)

NICU

a.How has your experience been in the NICU? Was it what you expected?

b. When thinking about the providers that are taking care of your baby, can you give me an instance or example when you felt supported or respected by the NICU team?

c. Are there examples or situations when you felt your baby’s providers were not helpful, treated you poorly, or your needs were not addressed? Please explain.

**IV.** MEDICAL DISCRIMINATION

***We have asked you many questions and talked about many different experiences with health care providers during your pregnancy, birth, and after the pregnancy. Would you like to take a break before this last section?***

***Now we are going to ask you questions about how you feel about the care you received generally across your pregnancy, birth, and after you left the hospital, rather than focusing on each of these time periods separately as we just did***

**1. Was your experience with the care you received what you had expected? Or not? Why?**

**2. In your interactions with care providers, what was your experience with…**

**a.** How they shared information with you. For example, answering your questions, explaining reasons for tests, and talking through your options for prenatal care and birth. Also ask: Are there things you had to find out on your own that would have been helpful for them to share with you?

**c.** The time they spend with you during appointments.

**b.** How compassionate and kind they were with you. For example, making you feel comfortable and like you could ask questions or voice concerns

**c.** How respectful and supportive they were with you.

**3. In your interactions with health care providers...**

a. Were they helpful? Or not? How? Can you say more? Do you have a sense of why?

b. Were the questions you asked taken seriously or were they dismissive of you? Or not? How? Can you say more? Do you have a sense of why?

c. Did you feel disrespected? Or not? How? Can you say more? Do you have a sense of why?

d. Did you feel treated badly/mistreated? Or not? How? Can you say more? Do you have a sense of why?

**4. Was the care you received different in some ways from the care that others you know have received from these providers? *If yes*, how?** a. (Interviewer: persistently probe here letting woman know we are interested in any thoughts she has about this.)

a. In your personal experience, do you feel you experienced any discrimination during your pregnancy?

b. Do you feel like your experience significantly affected your outcome of your delivery?

**c. Is there anything else you want to share with me about your experiences?**

***Thank you so much for sharing with me today about your experiences about your health care providers and experiences during your pregnancy, birth, and since you gave birth to ____________ (baby’s name). I appreciated hearing about your experiences and that you were willing to share them with me. I want to end by asking how it felt to you to talk about your experiences with me today? And, I am also wondering if you have any questions for me?***

Appendix B.

**Interpersonal Bias in Healthcare (Providers)**

We are conducting a brief survey about unconscious bias in the health care setting. Our goal is to ascertain medical providers’ knowledge and experience with unconscious bias and birth related post-traumatic stress disorder. Your responses are completely anonymous and will be aggregated. Please answer honestly.

Please indicate your role on the medical team: ____________________

(i.e. Faculty, Fellow, NNP, PA, RN)

1. Unconscious Bias can negatively impact patient care and perpetuate systemic inequalities.

Agree Unsure Disagree

1. There is sufficient evidence in the literature that suggests inequitable health care delivery contributes to disparities in perinatal outcomes.

Agree Unsure Disagree

1. Within the last 5 years, have you ever witnessed a patient getting substandard medical care and believe implicit bias played a role? If so, please give an example

Yes No

If YES, please provide context to this incident and decide what category would you classify this event?

____________________________________________________________

_____________________________________________________________

_____________________________________________________________

1. Offering treatment/interventions
2. Birth Planning/Delivery considerations
3. Communication
4. Deviation from Standard of Care Practices
5. Counseling

Postnatal post-traumatic stress disorder (PTSD) is a type of anxiety disorder. It is also known as birth trauma. Studies indicate birth-related PTSD impacts approximately 20% of postpartum patients while others may experience posttraumatic stress symptoms after birth but not meet the clinical criteria for PTSD.

1. In your opinion, what is the most common complaint from patients who reported a negative birth experience.

1. Treatment Decisions
2. Provider-patient interactions
3. Deviation from Birth Plan
4. Lack of Social Support
5. Prolonged Hospitalization of Mother or Infant
